# Genetic parameters for alternative resilience phenotypes in Holstein cows

**DOI:** 10.3168/jdsc.2025-0823

**Published:** 2025-10-03

**Authors:** Fiona Louise Guinan, Robert H. Fourdraine, Francisco Peñagaricano, Kent A. Weigel

**Affiliations:** 1 Department of Animal and Dairy Sciences, University of Wisconsin–Madison, Madison, WI 53706; 2 Dairy Records Management Systems, North Carolina State University, Raleigh, NC 27603

## Abstract

The ability of an animal to be minimally affected by or bounce back rapidly from a disturbance is known as resilience. With increasing variability in climate conditions, along with skilled labor shortages and disease outbreaks, the value of genetic selection of animals for increased resilience has become important. The goal of this study was to assess alternative resilience phenotypes in lactating Holstein cows. Our data set included 34,044 US Holstein cows from parities 1, 2, or 3 that experienced a single perturbation lasting at least 5 d but up to 14 d, with a severity of at least 5% for the entire duration of the perturbation. Perturbations were identified by detecting runs of poor performance at the pen level as the percentage difference between mean expected and mean observed milk production levels. After identifying the date within a detected perturbation with the lowest mean daily milk production level (nadir), we investigated 7 different resilience phenotypes for individual cows, comprising 2 distinct groups. The first consisted of 4 resilience phenotypes calculated using change in milk yield (ΔMY) over numerous days relative to the pen nadir. These phenotypes include (1) ΔMY throughout the entire event, (2) ΔMY before the nadir, (3) ΔMY near the nadir (1 d before, on the nadir, and 1 d after the nadir), and (4) ΔMY after the nadir. The second group of resilience phenotypes were calculated using a single day during the perturbation and include (5) residual on d 1 of the perturbation, (6) residual at the nadir, and (7) residual on the last day of the perturbation. Heritabilities for resilience phenotypes were estimated using a univariate model, whereas genetic correlations between resilience phenotypes and with 305-d milk yield were estimated using a bivariate model. Heritability (SE) estimates ranged from 0.004 (0.004) to 0.04 (0.009) for resilience phenotypes. Resilience traits calculated over the entire or partial perturbation period tended to show higher heritability estimates than those calculated using a residual on a single day. Genetic correlations among resilience indicators ranged from 0.21 (0.01) to 0.99 (0.01) and with 305-d milk yield ranged from −0.02 (0.45) to −0.30 (0.38), indicating potential to select for increased resilience while simultaneously increasing milk production. Overall, our findings suggest that resilience indicators based on subsets of the perturbation period or on single-day measurements tend to have low heritability. In contrast, resilience indicators calculated across several days within the perturbation period, such as ΔMY, capture greater genetic variation in resilience and are therefore more appropriate for the genetic improvement of resilience in dairy cattle.

## Highlights

• Resilience in cows is defined after identifying that a perturbation has occurred.
• Seven novel resilience traits were tested for 34,044 US Holsteins.
• ΔMY over the full perturbation period had the highest heritability for resilience.
• Single-day metrics were less informative for genetic selection.
• Genetic selection for ?MY can improve resilience while maintaining milk production.

## Graphical Abstract

**Figure:**
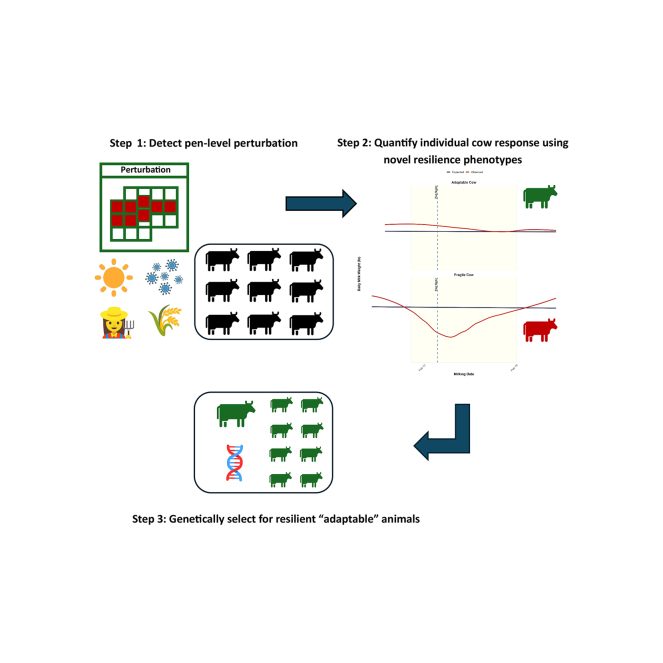
**Summary:** Resilience in dairy cattle refers to the ability to withstand or recover rapidly from environmental disturbances. With increasing climate and feed variability, labor shortages, and disease risks, improving animal resilience through genetic selection is critical. This study evaluated 7 alternative phenotypes of resilience for 34,044 US Holstein cows during a perturbation. Cows were grouped by their response to and recovery from the event, using measures of milk yield change (ΔMY) and milk yield residuals on specific days. Our results showed that resilience indicators calculated over the entire perturbation period, especially ΔMY, had higher heritability and more desirable genetic correlations with 305-day milk yield than single-day metrics. These findings suggest that ΔMY is a more informative phenotype for identifying genetically resilient cows. Selecting for this trait could help improve resilience in dairy herds while maintaining high productivity, making it a valuable tool for modern dairy farming.

Resilience has emerged as a key focus in the selection of animals that can maintain performance or be minimally impacted under increasing environmental perturbations (Colditz and Hine, 2016; Berghof et al., 2019). The goal of selecting for generalized resilience is to identify animals that remain healthy or respond minimally and recover rapidly in response to challenges such as extreme weather changes, variability in skilled labor availability, and feed shortages, while simultaneously maintaining high levels of milk production and improving animal health and welfare (Friggens et al., 2022). Generalized resilience is an animal's ability to be minimally affected by challenges, independent of the challenge experienced. Therefore, generalized resilience is challenging to measure, as it is difficult (without an experiment or introduction of a known perturbation) to identify a period during which all animals experienced the same challenge. To capture generalized resilience, Guinan et al. (2025) identified runs of poor performance at the pen level by quantifying the percentage difference between the expected and observed daily milk production. Expected milk production was estimated using a fourth-order polynomial fitted through quantile regression using a 0.5 quantile (Guinan et al., 2024). The exact cause of the perturbation leading to the runs of poor performance was unknown. This method was designed to identify the dates when mean observed milk production fell below mean expected production within the pen by a specified percentage for a defined number of consecutive days.

The foundational work to capture resilience indicators involved using the log-transformed variance of expected and observed daily performance using high-frequency data in different species (Putz et al., 2019; Poppe et al., 2020; Bedere et al., 2022). These indicators focused on the variance throughout the production period, without considering perturbations at either the group (pen) or the individual level. Hence, we define this trait as lactation consistency, as opposed to resilience (Guinan et al., 2024). The next step in developing resilience indicators is to dissect a perturbation into its response and recovery phases, providing a clearer picture of the animal's underlying biological function. This approach has been applied at the individual level, where perturbations unique to each cow are used to define their specific resilience profiles (Adriaens et al., 2021; Ben Abdelkrim et al., 2021; Wang et al., 2022).

For generalized resilience to perturbations that may or may not have been observed, we must first identify when such perturbations occur at the herd, group, or pen level. Guinan et al. (2025) used pen information attached to daily milk weights to retrospectively identify when a perturbation (or unknown challenge) occurred at the pen level. Then, individual cows' responses to the perturbation across multiple combinations of perturbation severity and duration were measured. In the present paper, we focused on 1 subset (≥5% severity and ≥5 d duration) to calculate alternative resilience phenotypes that can capture cows' response and recovery profiles during detected perturbations. Severity was defined as the percentage difference between mean observed and mean expected pen milk production, with a threshold of ≥5% in this subset. Duration referred to the minimum number of consecutive days the pen underperformed by ≥5%, which was set at a minimum of 5 d for this analysis.

The aim of the study was to assess alternative resilience phenotypes during runs of poor performance at the pen level, when there was evidence that the performance of the group was impaired but individual cows may have responded to the perturbation differently. Genetic parameters were estimated for alternative resilience phenotypes measured during the perturbation, including measures that represented performance on specific days within the perturbation and measures that spanned multiple days. In addition, genetic correlations between resilience phenotypes and 305-d milk production were estimated, to understand the relationship between alternative resilience phenotypes and lactation performance.

No human or animal subjects were used, so this analysis did not require approval by an Institutional Animal Care and Use Committee or Institutional Review Board. Data were provided by Dairy Records Management Systems (Raleigh, NC) and were extracted from the PCDART herd management software on their customers' farms. Our data set contained daily milk weights for 34,044 cows in lactation 1, 2, or 3 that experienced a perturbation with a minimum of 5% difference between expected and observed mean pen milk production for at least 5 d. Cows were required to have a record on (1) the first day, (2) the nadir, and (3) the last day of the perturbation. The nadir refers to the day during the perturbation when the pen's mean milk production reached its lowest point relative to the expected level—that is, the day with the greatest negative deviation between mean observed and mean expected pen milk production. Perturbations were detected using runs of poor performance as described in Guinan et al. (2025). The data set contained 13,701 primiparous cows, 12,655 second-parity cows, and 7,688 third-parity cows. All cows experienced only 1 perturbation event, which was the most severe event. The majority of events lasted 5 d total (34%), with events lasting an average of 6.7 d (SD 1.3 d).

Expected milk production values were calculated using a fourth-order polynomial and quantile regression using a 0.5 quantile (Guinan et al., 2024). The first step in the analysis was to identify the pen nadir (i.e., when milk production reached its lowest level during the perturbation) based on mean observed daily milk production at the pen level (Figure 1a). Once the nadir was identified, we calculated 7 different resilience phenotypes. These were split into 2 distinct groups. The first group consisted of 4 resilience phenotypes calculated using change in milk yield (**ΔMY**) over numerous days relative to the pen nadir (Figure 1a). These phenotypes include (1) ΔMY throughout the entire event, (2) ΔMY before the nadir, (3) ΔMY near the nadir (1 d before, on the nadir, and 1 d after the nadir), and (4) ΔMY after the nadir (Figure 1a). Each of the phenotypes were calculated using the following formulas:

$$\mathrm{Mean}\mathrm{residual}\mathrm{milk}\mathrm{productio}n_{ij}\left(\mathrm{kg}\right)=\frac{\sum (\mathrm{yiel}d_{ij}-{\hat{\mathrm{yield}}}_{ij})}{n_{ij}};$$

$$\mathrm{Mean}\mathrm{expected}\mathrm{milk}\mathrm{productio}n_{ij}\left(\mathrm{kg}\right)=\frac{\sum ({\hat{\mathrm{yield}}}_{ij})}{n_{ij}};$$

$$\Delta MY_{ij}\left(\%\right)=\frac{\mathrm{Mean}\;\mathrm{residual}\;\mathrm{milk}\;\mathrm{production}{\;}_{ij}\;}{\mathrm{Mean}\;\mathrm{expected}\;\mathrm{milk}\;\mathrm{production}{\;}_{ij}}\times 100,$$

where *i* is cow, *j* is the event, and *n* is the number of records per cow within the perturbation period.

**Figure 1:**
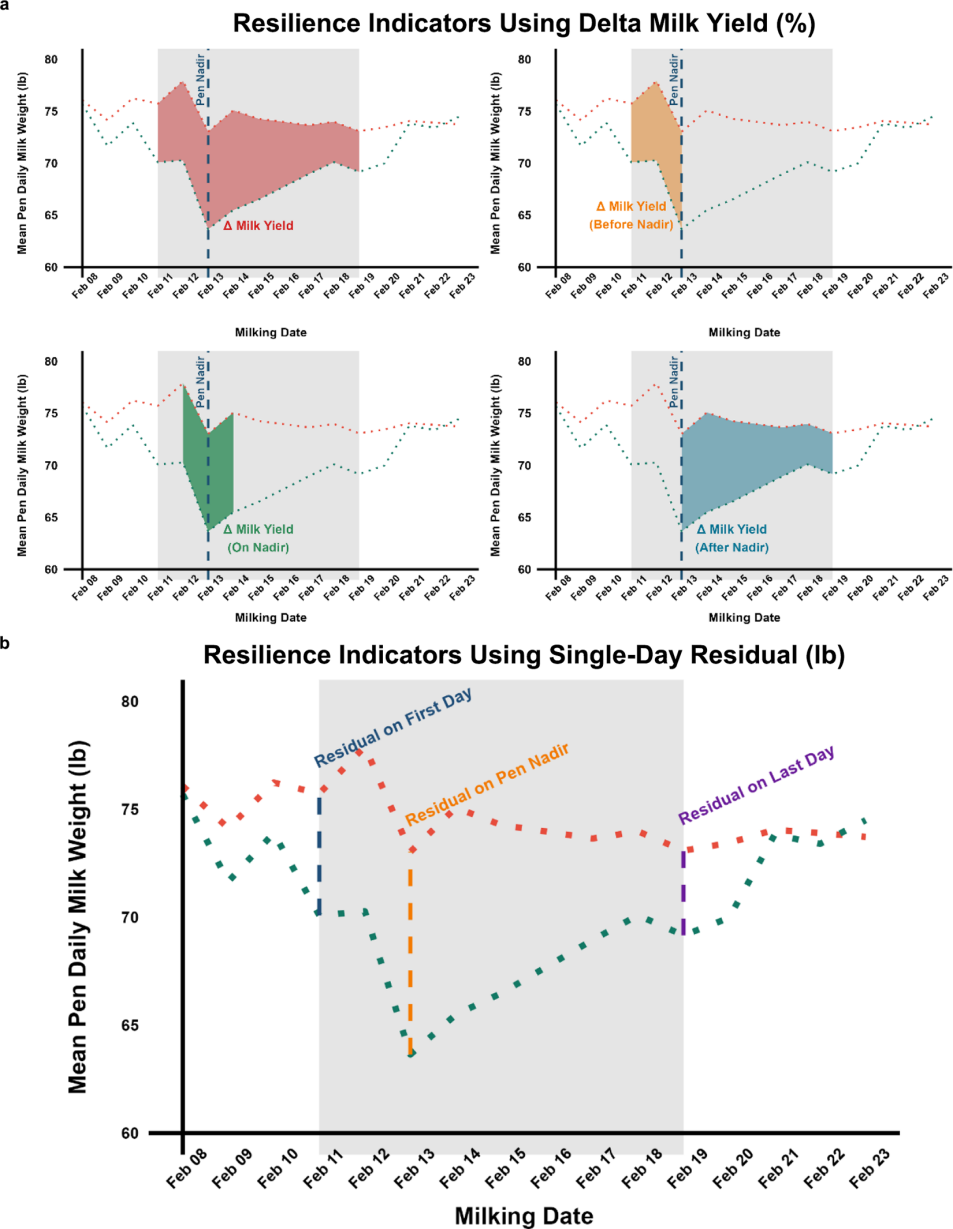
Perturbation within a pen detected using “runs of poor performance” methodology (see Guinan et al., 2025). The green dotted lines show the actual mean pen daily milk weight, and the orange dotted lines represent the expected mean pen daily milk weights. The gray sections represent the perturbation period, where there is at least a 5% difference between observed and expected mean pen daily milk weight for a minimum of 5 d (Feb. 11–Feb. 19, 2024). (a) Each of the 4 resilience phenotypes (ΔMY and ΔMY before, near, and after the nadir), calculated using data spanning multiple days. (b) Each of the 3 resilience phenotypes on the first day, at the nadir, and on the last day of the perturbation, were calculated using a single residual. Nadir refers to the day during the perturbation when the pen's mean milk production reached its lowest point relative to the expected level (i.e., the day with the greatest negative deviation between mean observed and mean expected pen milk production).

The second group of resilience traits were calculated using a single day during the perturbation and included (5) residual on the first day of the perturbation, (6) residual at the nadir, and (7) residual on the last day of the perturbation (Figure 1b). These phenotypes were calculated as

$$\mathrm{Residual}\mathrm{milk}\mathrm{productio}n_{ij}\left(\mathrm{kg}\right)=\frac{\;\left(\mathrm{yiel}d_{ij}-{\hat{\mathrm{yield}}}_{ij}\right)\;}{{\hat{\mathrm{yield}}}_{ij}},$$

where *i* is cow and *j* is the event.

Variance components and heritability estimates were calculated for the 7 resilience phenotypes using the following univariate animal model:

*y_ijklmno_* = CA*_i_* + DIM*_j_* + Parity*_k_* + HYS*_l_* + event*_m_* + a*_n_* + *e_ijklmno_*,

where *y_ijklmno_* is the resilience phenotype (% or kg), CA*_i_* is the fixed effect of calving age with 18 levels, DIM*_j_* is the days in milk per cow on the first day of the perturbation period with 10 levels, Parity*_k_* is the fixed effect of lactation with 3 levels, HYS*_l_* is the fixed effect of herd year season (minimum of 5 observations per level; 813 levels), event*_m_* is the random effect of the perturbation period the cow experienced (minimum of 25 observations per level; 588 levels), a*_n_* is the random effect of cow modeled using up to 5 generations of pedigree data (34,044 levels) distributed as $a\&sim;N\left(A\sigma_{a}^{2}\right),$ and *e_ijklmno_* is the random residual effect (subscript *o* represents the random residual effect) distributed as $e\&sim;N\left(I\sigma_{e}^{2}\right).$ **A** is the numerator relationship matrix, and **I** is the identity matrix. Genetic correlations among resilience phenotypes and with 305-d milk production were estimated using bivariate models. All analyses were performed using AIREMLF90 software (Aguilar et al., 2018). It should be noted that for all of the phenotypes, a positive value indicates a more resilient animal. For example, in the first group of phenotypes, a positive ΔMY, independent of the period, represents an animal that did not experience any milk loss throughout the perturbation. Similarly, in the second group of phenotypes, or the residuals, a positive residual indicates that the animal performed above expected on that particular day during the perturbation.

Heritabilities for resilience phenotypes investigated during the perturbation ranged from 0.004 (0.004) to 0.04 (0.009; Table 1). Estimated heritability for overall ΔMY was the highest, perhaps because it spanned the greatest number of days and its mean would be expected to have the smallest SE. Heritability estimates were lowest for the residual on d 1 of the perturbation period, 0.008 (0.004), and the last day of the perturbation period, 0.004 (0.004). Heritability estimates before, near, and after the nadir were 0.02 (0.006–0.007), and, interestingly, the heritability estimate for a single residual on the nadir was also 0.02 (0.007; Table 1). Although the residual at the nadir was computed from a single daily milk record, its heritability estimate was comparable to the resilience phenotype calculated as the mean of 3 daily records near the nadir, perhaps because the additive genetic variance was greatest on the most challenging day of the perturbation (Table 1). Our heritability estimates were lower than heritabilities calculated using individual-level perturbations for resilience (Wang et al., 2024), and this could be partially attributed to the fact that, in that analysis, an individual-level perturbation was required to be a minimum of 10 d, with observed milk production 90% below the expected value at least once, which may have decreased the possibility of false positive perturbations in the final data set. Guinan et al. (2025) previously showed that heritability estimates for resilience tended to increase as the severity and duration of perturbations increased, although data sets were smaller and SE of estimated genetic parameters were larger as perturbation definitions became stricter.

**Table 1:** Summary statistics, phenotypic means (SD), variance components, and heritability estimates (SE) for 7 resilience phenotypes measured during a perturbation event^1^

Phenotype	Cows (n)	Events (n)	Phenotypic mean (SD)	$\sigma_{event}^{2}$	$\sigma_{a}^{2}$	$\sigma_{e}^{2}$	h^2^
Δ Milk yield overall (%)	34,044	588	−8.8	3.470	1.811	40.225	0.04
			(7.2)	(0.371)	(0.389)	(0.459)	(0.009)
Δ Milk yield before nadir (%)	34,044	588	−9.7	8.134	2.00	82.735	0.02
			(9.8)	(0.839)	(0.551)	(0.807)	(0.006)
Δ Milk yield near nadir (%)	34,044	588	−9.9	8.986	1.987	84.527	0.02
			(9.9)	(0.911)	(0.562)	(0.825)	(0.006)
Δ Milk yield after nadir (%)	34,044	588	−12.2	9.279	2.633	98.617	0.02
			(11.1)	(0.971)	(0.778)	(0.778)	(0.007)
Residual on d 1 (kg)	34,044	588	−0.036	0.0009	0.00009	0.01	0.008
			(0.073)	(0.00009)	(0.00005)	(0.00009)	(0.004)
Residual at nadir (kg)	34,044	588	−0.05	0.0004	0.0001	0.004	0.02
			(0.05)	(0.00004)	(0.00004)	(0.00005)	(0.007)
Residual on last day (kg)	34,044	588	−0.03	0.0001	0.00002	0.003	0.004
			(0.04)	(0.00002)	(0.00001)	(0.00003)	(0.004)

1 $\sigma_{event}^{2}$ = event variance; $\sigma_{a}^{2}$ = additive genetic variance; $\sigma_{e}^{2}$ = residual variance; h^2^ = heritability, nadir = the day during the perturbation when the pen's mean milk production reached its lowest point relative to the expected level (i.e., the day with the greatest negative deviation between mean observed and mean expected pen milk production).

Genetic correlations between resilience phenotypes and with 305-d milk yield were estimated using bivariate models to understand the relationship between resilience indicators and their association with milk production. The genetic correlations among resilience indicators ranged from 0.21 (0.01) to 0.99 (0.02). The highest genetic correlations were observed between the phenotypes calculated over multiple days during the perturbation period (i.e., ΔMY phenotypes). As expected, the estimated genetic correlations between resilience phenotypes calculated over multiple days and those calculated on single days were lower and ranged from 0.21 (0.01) to 0.87 (0.01), indicating that ΔMY and single-day residual phenotypes reflect different biological functions underlying how animals respond to and recover from perturbations.

Genetic correlations between resilience traits and 305-d milk yield ranged from −0.30 (0.38) to −0.02 (0.45; Table 2). The SE for the estimated genetic correlations between resilience indicators and 305-d milk yield were highest for the resilience phenotypes calculated using a single residual on either the first day, the nadir, or the last day of the perturbation (Table 2). All the genetic correlations were negative, indicating that as milk yield increases, resilience decreases. However, the genetic correlations were low, especially for overall ΔMY, indicating we can select cows that are both resilient and high producing.

**Table 2:** Genetic correlations (SE) between resilience phenotypes and with 305-d milk yield (kg)^1^

Item	Δ Milk yield overall (%)	Δ Milk yield before nadir (%)	Δ Milk yield near nadir (%)	Δ Milk yield after nadir (%)	Residual on d 1 (kg)	Residual at nadir (kg)	Residual on last day (kg)	305-d milk (kg)
Δ Milk yield overall (%)	1	0.99 (0.02)	0.99 (0.01)	0.97 (0.03)	0.92 (0.25)	0.98 (0.07)	0.88 (0.08)	−0.13 (0.09)
Δ Milk yield before nadir (%)		1	0.99 (0.02)	0.92 (0.09)	0.94 (0.50)	0.94 (0.06)	0.97 (0.14)	−0.22 (0.11)
Δ Milk yield near nadir (%)			1	0.98 (0.03)	0.90 (0.30)	0.97 (0.05)	0.98 (0.12)	−0.20 (0.11)
Δ Milk yield after nadir (%)				1	0.67 (0.40)	0.87 (0.01)	0.78 (0.01)	−0.07 (0.12)
Residual on d 1 (kg)					1	0.66 (0.34)	0.21 (0.01)	−0.30 (0.38)
Residual at nadir (kg)						1	0.41 (0.01)	−0.06 (0.12)
Residual on last day (kg)							1	−0.02 (0.45)
305-d milk (kg)								1

1 Nadir = the day during the perturbation when the pen's mean milk production reached its lowest point relative to the expected level (i.e., the day with the greatest negative deviation between mean observed and mean expected pen milk production).

The main goal of this research was to define precise resilience indicators that reflect the biological functioning of a cow using daily milk weights during a perturbation, such that we can estimate the degree to which these traits are under genetic control and assess their suitability as resilience phenotypes that can be improved through selection. It is important to note that we currently do not have a gold standard for measurement of resilience, because detailed farm-level or pen-level information about management or environmental disturbances are lacking, and we must therefore infer the occurrence, duration, and severity from the data. Furthermore, we do not know the physiological functions associated with resilience to external perturbations, and we must therefore assess their genetic correlations with other important traits, such as longevity, fertility, and health resistance (Friggens et al., 2023). Our data set contained cows that experienced a single event, as we kept only the worst perturbation to which each cow was exposed; the relatively small number of cows exposed to multiple perturbations precluded including repeated records and estimating permanent environmental effects. We chose the combination of ≥5% severity and ≥5 d perturbation from options considered by Guinan et al. (2025) to achieve a balance between too many false-positive perturbation events (due to criteria too lax) and too few cows in the data set (due to criteria too strict). We investigated 7 different phenotypes that reflected different stages of perturbation relative to the pen nadir (i.e., the worst day of the mean performance for all cows in the group).

The resilience phenotypes investigated in this analysis capture the performance of cows at different points within an identified perturbation—including the response phase (before the nadir), the most challenging phase (at or near the nadir), and the recovery phase (after the nadir). These resilience phenotypes have been explored previously using simulated data (Ghaderi Zefreh et al., 2024). However, field data present many additional challenges, beyond the obvious limitation that the true onset, severity, and duration of perturbation are unknown. In dairy herds, for example, daily milk yield data are noisy and sometimes missing, and cows frequently move between pens. As a result, it is difficult to identify perturbations and define distinct phenotypes that describe each animal's response to the disturbance. By using an approach based on runs of poor performance to identify perturbations occurring at the pen level, we can ensure that all cows within the pen experience the same microenvironment. This methodology may be more effective in capturing environmental perturbations that affect all animals in the pen at the same time, such as extreme weather events and feed anomalies, as opposed to perturbations such as disease outbreaks, in which individual cows may be exposed to a pathogen on different days. Additionally, our findings highlight the need for multiple data points for each animal during a perturbation event, to truly capture how each animal responds to and recovers from an exogenous challenge.

Our findings indicate that ΔMY over the entire perturbation seems to be the most promising phenotype for identifying and selecting resilient cows. Overall ΔMY throughout the perturbation captures both the response and recovery phases and provides a more comprehensive profile of a cow's performance during the entire challenging period. That said, it may be useful to incorporate this novel resilience phenotype into a selection index along with lactation consistency (TempVar; log-transformed variance between observed and expected milk production throughout the entire lactation period) to facilitate the selection of animals that are consistent, resilient, and robust in the face of increasingly frequent perturbations.
